# A Treatise on Operative Surgery, Comprising a Description of the Various Processes of the Art, Including All the New Operations; with 80 Plates, Containing 486 Separate Illustrations

**Published:** 1844-07

**Authors:** 


Art. VI.—*A Treatise on Operative Surgery, comprising a description of the various processes of the art, including all the new operations;* exhibiting the state of Surgical Science in its present advanced condition; with 80 plates, containing 486 separate illustrations. By Joseph Pancoast, M. D., Professor of General, Descriptive, and Surgical Anatomy in Jefferson Medical College, Philadelphia, &c., &c. Philadelphia: Cary & Hart, for G. N. Loomis. 1844. pp. 380. 4to.

Here is a great book—not in that sense in which it was said, a *great book is a great evil*—but a work of great size, of excellent design, and splendid execution. The labor of preparing such a work could not have been else than great, and its value to the profession may be pronounced very great. Better judges than we profess to be in such matters, declare, that the engravings surpass in beauty and accuracy anything that the American arts have yet produced in that line.

Of the opportunities for making his work what it purports to be, Dr. Pancoast thus speaks in the advertisement:

"With these admirable treatises (that of Froriep, of Berlin, and that of Bougery, of Paris,) before him as a guide, and having at hand the greater portion of the surgical works, which have recently appeared in various languages, and with the advantage which nine years' continuous service in one of the largest hospitals of North America has given him, not only in comparing to a certain extent the value of the different methods, but in enabling him to obtain a large number of accurate drawings of operations which have been done by his own hand, the author has endeavored to furnish a work that shall represent, so far as its limits will allow, the operative surgery of the day."

It is stated in the advertisement, that the limits proposed in the prospectus were found, as the author progressed, to be too restricted for so copious a subject, and that the publishers, with great liberality, consented to add sixteen plates, and one hundred and thirty pages of description, to the number at first promised, without increase of price to the subscribers. A worthy gentleman acting as agent for this work spent some weeks in our city, last winter, on his way to the South. We hope he met with liberal encouragement. By patronizing such works we are rendering a substantial service to our profession.

The examiner in his inspection of these drawings sees all the parts as they would appear to him if engaged in the operation. But besides the instruction thus clearly imparted by this admirable contrivance, the work contains a brief but sufficiently extended description of the surgical anatomy of the parts immediately concerned in the various operations. The pathological changes are also noticed where any account of these appeared necessary. The therapeutical management of surgical affections is not given at much length, because that would have extended the work to an immoderate size. It would hardly be expected in a work on operative surgery. Dr. Pancoast has done justice to the discoveries and improvements of American surgeons, and we hazard nothing in saying, that his work will enhance the character of the American profession wherever it appears. Y.

